# Machine Learning Analysis and Monomer Screening of Polyamide Nanofiltration Membranes for Lithium Separation

**DOI:** 10.1021/acsestengg.5c00444

**Published:** 2025-10-29

**Authors:** Raghav Dangayach, Nohyeong Jeong, Yongsheng Chen

**Affiliations:** School of Civil & Environmental Engineering, 1372 Georgia Institute of Technology, Atlanta, Georgia 30332, United States

**Keywords:** lithium, membranes, nanofiltration, machine learning, material screening, and inverse design

## Abstract

Nanofiltration (NF) membranes are increasingly being used to achieve precise solute–solute separation. These membranes are commonly synthesized using interfacial polymerization, offering great potential to separate lithium from magnesium. In this study, we have developed machine learning models that relate fabrication conditions, membrane properties, and operational conditions of NF membranes to predict water permeability and lithium/magnesium selectivity. Morgan fingerprints (MFs) and molecular descriptors (MDs) are used to represent the chemical and physical properties of the monomers. Explainable artificial intelligence tools such as Shapley additive explanations (SHAP) and partial dependence plots are used to evaluate the effects of the synthesis conditions and membrane properties on membrane performance. Based on the insights obtained from SHAP analysis, we developed a material screening approach to find promising monomers from a list of amines and cation-based ionic liquids. We construct a reference MF using the functional groups that positively contribute to membrane performance and compute a screening score that favors potential candidates with more desirable MDs. Finally, the synthesizability of these monomers is assessed using the synthetic accessibility score to find the most promising candidates. We compared the performance of screened monomers against traditional ones to validate the reliability of our approach. The results of this study provide critical insights into the relationships between synthesis conditions, membrane properties, and performance and establishes a novel, strategic framework for rational screening of monomers for NF membrane synthesis. This approach holds promise to accelerate the discovery of high-performance membranes tailored for specific separation challenges, thereby advancing the field of membrane technology.

## Graphical abstract

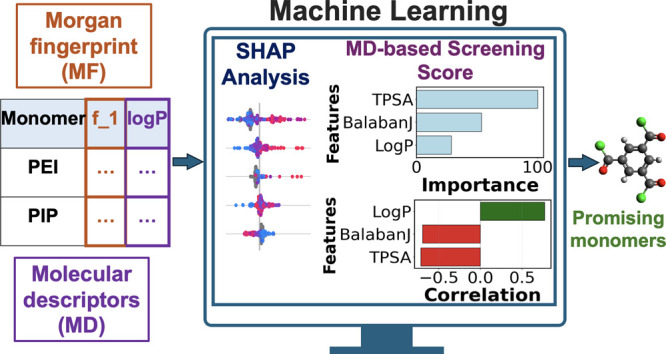

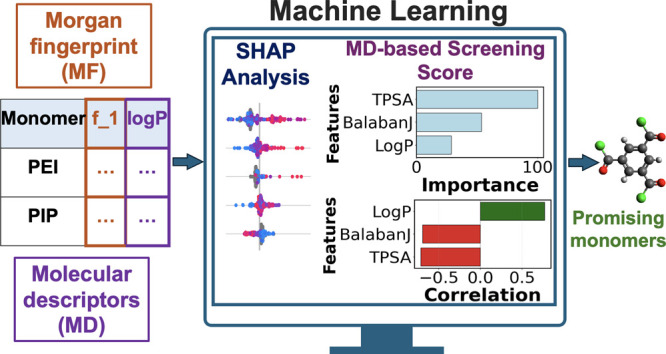

## 1 Introduction

The rapid rise in industrialization and global warming has led to an increased demand for cleaner energy production. In this context, lithium (Li) has become a critical metal since it is essential to produce rechargeable metal-ion batteries commonly used in electric vehicles, portable electronics, and grid-scale energy storage systems.^1,2^ This growing demand for Li has intensified global interest in efficient recovery methods from natural resources such as mineral ores and salt-lake brine. Conventional mining practices used to recover Li from mineral ores pose a threat to the natural ecosystem due to large levels of toxic waste being released into the environment.^3^ It has been estimated that over 2.3 × 10^11^ t of Li is found in natural water bodies, making its extraction and recovery a highly desirable area of research and development.^4^

Techniques such as solar evaporation, ion exchange, and chemical precipitation are commonly used to recover Li from saline water.^5,6^ These techniques present challenges from a chemical and environmental engineering standpoint. Ion exchange is complicated due to exhaustion of resins at highly concentrated metal brines. On the other hand, postrecovery in solar evaporation and chemical precipitation is complex due to the existence of co-ions such as sodium, potassium, magnesium (Mg), and calcium. Mg specifically poses a great difficulty in Li recovery since it coprecipitates with Li as MgCO_3_.^7^ As a result, these traditional techniques are more suitable for brines with a low Mg-to-Li ratio (MLR).^8^ Nanofiltration (NF) is a pressure-driven membrane technology that demonstrates promising performance in the separation of Li and Mg in liquid streams with high MLR.^9–11^ NF has several advantages over conventional methods, including high selectivity, scalability, and good process and energy efficiency, leading to improved engineering outcomes.^12^ Interfacial polymerization (IP) is widely used to fabricate NF membranes by reacting an amine monomer in the aqueous phase with an acyl chloride monomer in the organic phase, forming a thin polyamide (PA) layer on top of a porous substrate.^13^ The physical and chemical properties of this thin PA film can be tuned by selecting different types of monomers, incorporating additives (e.g., nanomaterials and surfactants), or adjusting the synthesis conditions, such as reaction time, heat curing time, and heat curing temperature.^14–17^ An in-depth understanding of these synthesis-property-performance relationships is critical to the design of high-performance PA-NF membranes for Li separation.

The commonly used metrics to evaluate the performance of PA-NF membranes for Li separation are water permeability and Li/Mg selectivity. These two metrics exhibit a well-documented trade-off due to the intrinsic material limitations wherein more permeable membranes are less selective and vice versa.^18^ This inherent trade-off continues to drive efforts toward the development of advanced membranes that achieve high water permeability and salt selectivity. Achieving this goal requires innovative strategies capable of efficiently navigating the expansive design space, which includes diverse types of monomers and several combinations of synthesis conditions.^19^ The traditional trial-and-error approach of membrane synthesis often requires multiple design cycles to fabricate high-performance membranes. It is impractical for humans to explore this large design space through iterative experimentation due to its expensive and cumbersome nature.^20^

Machine learning (ML) is a data-driven approach that has made significant advances in the field of membrane science and technology.^21–24^ By leveraging experiments conducted over the past few decades, ML enables the development of powerful tools to address complex, multidimensional problems, while accurately predicting membrane performance and properties.^25–27^ Researchers have used ML to predict water permeability, salt rejection, fouling decline ratio, and flux recovery ratio based on their synthesis conditions and properties.^28^ However, due to the “black box” nature of these ML models, it is challenging to understand the decision-making process behind the predictions made by them.^29^

Explainable artificial intelligence (XAI) has recently emerged as a critical framework to elucidate the rationale behind the decision-making process of ML models.^30^ Shapley additive explanations (SHAP) and partial dependence plots (PDPs) are commonly used XAI tools that can reveal the impact of various features on the ML models, providing insights into model predictions.^31,32^ Deng et al. used univariate and bivariate PDPs to investigate the influence of synthesis conditions of PA membranes critical to achieving high selectivity in solute–solute separation.^33^ Jeong et al. implemented SHAP to understand the underlying features governing ion transport across PA-NF and reverse osmosis (RO) membranes.^29^ By unveiling the impact of polymer properties, synthesis conditions, and the underlying transport mechanisms, XAI not only guides the membrane design process but also facilitates the discovery of new materials for specific applications.^20^ The widely used approach for material screening is to implement SHAP analysis to capture key chemical functional groups and screen promising candidates through similarity metrics such as the Tanimoto coefficient.^34^ This approach has proven effective; however, it overlooks the structural and physical characteristics of the polymer within the screening process. Gong et al. demonstrated the importance of using such molecular descriptors (MDs) while representing monomers to develop predictive ML models for the synthesis of PA-NF membranes.^35^ Thus, inclusion of these MDs in the material search process will streamline the screening pipeline and assist in the identification of novel candidates for NF membrane synthesis.

Some previous studies provide an ML perspective relating membrane properties and operational conditions to understand Li separation.^9,36–38^ Our work builds on the existing literature by using data-driven tools to develop synthesis-property-performance relationships to improve the overall understanding of PA-NF membranes for Li separation and to devise a screening methodology that accelerates the discovery of high-performance materials for membrane synthesis. In this study, we have curated a database containing synthesis conditions, membrane properties, and membrane performance through literature mining. The primary objective of our study is (1) to develop predictive ML models relating the input features to performance metrics such as permeability and Li/Mg selectivity, (2) to study the impact of synthesis conditions and membrane properties on its separation performance using XAI tools, and (3) to develop high throughput virtual screening setup combining both Morgan fingerprints (MFs) and MDs of monomers to screen promising candidates with the potential for superior membrane performance.

## 2 Materials and Methods

### 2.1 Data Collection and Data Featurization

The data set for this study was collected from different sources and publishing aggregators such as Google Scholar, Elsevier, and ACS. The data exclusively focused on PA-NF membranes synthesized using IP for Li/Mg separation. The input data matrix consisted of 19 variables that were split into 3 categories: (1) synthesis conditions, (2) membrane properties, and (3) operational conditions (Table S1, Supporting Information). Membrane properties were represented using surface hydrophilicity (water contact angle), membrane pore characteristics (pore size), and surface charge (zeta potential). The values collected for the features were exhaustively mined from tables and text. For data from graphs, WebPlotDigitizer was used to extract graphical data.^39^ The final data set for our study consisted of 256 data points for water permeability (LMHbar^–1^) and 215 data points for Li/Mg salt selectivity. Importantly, we only considered the publications that reported selectivity values measured from systems containing multiple salts (containing both LiCl and MgCl_2_) as feed in lieu of systems that measure selectivity in 2 different systems containing a single salt (consisting either of LiCl or MgCl_2_).^40^ This criterion was applied because multi-ion systems represent the salt selectivity more accurately compared to singular ion systems and give a better representation of the separation process between Li and Mg.

The data set consisted of both numerical and categorical features, and no missing data were imputed since every feature has its physicochemical contribution to the membrane performance.^41^ Amine monomers used to synthesize NF membranes are reported in publications as a categorical variable i.e., names such as piperazine (PIP) or polyethylenimine (PEI). For better representation of these monomers for ML model training, we generated additional features that allow us to capture their chemical and physical attributes. To consider the chemical structure of monomers/polymers, we used the simplified molecular-input line-entry system (SMILES) and converted them into MFs using the RDKit package.^42^ The presence and absence of a specific atomic group/structural feature of molecules is encoded as 1 and 0, respectively. Additionally, we also used MDs to capture the various structural, chemical, electronic, and topological properties of the monomers (Table S2, Supporting Information).

### 2.2 ML Model Development and Characteristics

ML models such as Random Forest (RF), XGBoost, CatBoost, and LightGBM have showcased strong capabilities, providing good prediction accuracy with superior interpretability for evaluating membrane performance.^34,43^ To prepare the data for model training, StandardScaler was used to scale the numerical feature values, and one-hot encoder was applied to encode categorical variables. We used Bayesian optimization with 5-fold cross-validation to develop ML models and identify the optimal hyperparameters for each ML algorithm (Table S3, Supporting Information). After these hyperparameters were optimized, we retrained the models to obtain the final performance metrics. For model training, 80% of the data set was used, and the remaining 20% of unseen data was set apart for testing. A shuffle split cross-validation strategy with 5 splits was implemented to ensure model generalizability and reduce the risk of overfitting. This resampling process was repeated across 8 random seed initializations, and the average performance metrics were computed to ensure robustness. The performance metrics being tested were coefficient of determination (*R* ^2^), root mean squared error (RMSE), and mean absolute error (MAE).

### 2.3 Model Interpretation Using XAI

Due to the inherent “black box” nature of ML models, it is challenging to explain the decision-making process behind their predictions. XAI can help humans understand how ML models are making their decisions and, more importantly, how certain input features influence model output.

#### 2.3.1 PDPs

PDP is a graphical representation illustrating the relationship between specific features and the predicted outcome of a model while keeping all other features constant. They help in understanding the marginal effects of a single or multiple features on membrane performance.^44^ We constructed single-variable PDP (capturing the effect of a single feature) and multivariable PDP (capturing the effect of two variables) to visualize the impact of the input variables on water permeability and Li/Mg selectivity. The average partial dependence function for a feature S, *f*_s_ can be calculated using

1 $$f_{s}=E_{x_{C}}\left[f\left(x_{s},x_{c}\right)\right]=\int f\left(x_{s},x_{c}\right)dP\left(x_{c}\right)$$

where feature C is the complement of S; *x*_c_ and *x*_s_ are their feature vectors, respectively.^45^

#### 2.3.2 SHAP

SHAP analysis is based on cooperative game theory, which is used to explain the contribution of each input variable to the model’s output.^46,47^ By calculating marginal contribution of each feature, SHAP analysis can reveal whether input variables have positive or negative impact on model output. It was also used to represent the changes in the membrane permeability and salt selectivity over the range of input feature. The SHAP values for an input feature *x* (of *n* total features) give prediction p as

2 $$\phi_{x}\left(p\right)=\sum_{S\subseteq N\backslash x}\frac{\left|S\right|!\left(n-\left|S\right|-1\right)!}{n!}\left(p\left(S\cup x\right)-p\left(S\right)\right)$$

where *S* is the subsets of all features with feature *x*, *p*(*S* ∪ *x*) are the predictions by the built ML model considering feature *x*, and *p*(*S*) are the predictions without considering feature *x*. The differences among all possible subsets of *S* ⊆ *N*\*x* are calculated due to the dependency of the effect of withholding a feature on other features in the ML model.^48^

### 2.4 Potential Monomer Screening

We developed a 3-step screening process to identify monomers for the synthesis of high-performance membranes with exceptional solute–solute performance. Over 500 candidate monomers were used to find the best candidates, which were obtained from the National Institute of Materials Science (NIMS) materials database and research publications focused on ionic liquids; a new class of candidates showing promise in the synthesis of membranes showing exceptional Li/Mg separation performance.^49–51^

#### 2.4.1 Reference MF Using the Tanimoto Coefficient

Based on the results obtained from SHAP analysis of input MFs, we constructed a reference MF by adding the chemical functional groups having positive contributions to membrane permeability and salt selectivity. Using this reference MF, we aim to find compounds with similar chemical properties since they will show greater potential for the synthesis of high-performance NF membranes. We calculated the Tanimoto coefficient (S_A,B_) to find the similarity between a candidate monomer and the reference. This is computed as the number of bits in common divided by the total number of bits, represented as

3 $$S_{A,B}=\frac{c}{a+b-c}$$

where *a* is the number of bits in molecule A, *b* is the number of bits in molecule B, and *c* denotes the number of bits that are in both molecules. Tanimoto coefficient of 1 represents an identical molecule, whereas 0 represents no similarity between the molecules.^34^

#### 2.4.2 Screening Score Using MDs

After the chemically similar monomers to the reference MF were obtained from the candidate database, we subjected the monomers to a screening test based on the relative impact of each MD on the ML models. This impact was a product of the relative importance of the MD to model output and the correlation of its SHAP values to permeability and Li/Mg selectivity. Once this impact was calculated for both models, the MD values of potential monomer candidates were scaled based on it. The final metric is referred to as the screening score (S.S.), which is calculated as

$$\mathrm{screening score}(S.S.)=\sum {\mathrm{feature impact}}_{p}x_{i}+\sum {\mathrm{feature impact}}_{s}x_{i}$$ 4

where feature impact_p_ and feature impact_s_ refer to the product between the SHAP importance and Spearman’s correlation of the SHAP values of the MD to the ML model for permeability and Li/Mg selectivity, respectively. *x*_*i*_ refers to the MD value of the potential monomer. The SHAP importance and correlation values for the best-performing ML model are calculated for 8 random seeds. The median values of importance and correlation for each descriptor across the random seeds are used as the final value to compute the feature impact and the resulting screening score. The idea is to ensure that candidate monomers having desirable properties score higher as compared to the other monomers, which will aid in the search for better candidates for NF membrane synthesis.

#### 2.4.3 Synthetic Accessibility Score (SAS)

In addition to using reference MF and scaled MD values for the screening process, we calculated the SAS of candidate monomers to find the hypothetical ease of synthesizing certain monomers compared to others.^52,53^ SAS ranges from 1 (easily synthesizable) to 10 (very difficult to synthesize). We used RDKit for the computation of the SAS of the potential monomeric candidates.

### 2.5 Membrane Fabrication and Experimental Validation

Three screened amine candidates, 2-methylpiperazine (M-PIP), 1-(2-aminoethyl)­piperazine (A-PIP), and trans­(2,5)-dimethylpiperazine (T-PIP), and two commonly used monomers, i.e., PEI (MW–800 Da) and PIP, were used to fabricate PA-NF membranes. The membranes were fabricated at modal conditions obtained from the data set. In short, a 0.5 wt % solution of monomer A1 was dissolved in water and allowed to contact a poly­(ether sulfone) (PES) substrate. The excess solution was removed from the surface of the substrate using a rubber roller. 0.1 wt % solution of trimesoyl chloride (TMC) and *n*-hexane was then poured onto the substrate and allowed to react for 60 s. The resultant membrane was cured at a temperature of 60 °C for 10 min to allow the formation of a thin PA layer. The resultant membrane was stored in DI water for further use.

NF membrane performance experiments were conducted using a membrane module consisting of a crossflow testing cell with an effective area of 4.1 cm^2^ and a flow rate of 450 g/min. The membranes were compacted at 8 bar for 1 h before running the tests. The water permeability of each membrane was calculated as

$$\mathrm{water permeability}=\frac{V}{A\times \Delta T\times P}$$

where *V* is the volume of water collected (*L*), *A* is the effective filtration area (m^2^), Δ*T* is the time interval (*h*), and *P* is the operation pressure (bar).

To calculate the separation performance of Li with respect to Mg, a salt solution was prepared by mixing appropriate amounts of MgCl_2_ and LiCl to achieve the desired mass ratio of 20:1. The membrane performance for solute–solute separation can be quantified using a selectivity factor *S*_Li/Mg_.

$$S_{\mathrm{Li}/\mathrm{Mg}}=\frac{\frac{C_{\mathrm{Li},p}}{C_{\mathrm{Li},f}}}{\frac{C_{\mathrm{Mg},p}}{C_{\mathrm{Mg},f}}}$$

Here, *C*_Li,p_, *C*_Li,f_ and *C*_Mg,p_, *C*_Mg,f_ are the concentrations of Li and Mg in permeate and feed, respectively.^54^ The concentration of the Li and Mg ions in the solution was analyzed using ICP-OES (PerkinElmer AA900Z).

## 3 Results and Discussion

### 3.1 Data Description, Analysis, and ML Model Performance

In Figure S1 (Supporting Information), we observe the permeability-selectivity trade-off across the entire data set, comparing membranes with additives and modifications to pristine NF membranes. Figures S2 and S3 (Supporting Information) present the data characteristics of the numerical and categorical features of the data set. The most commonly used amine monomers/polymers are PEI and PIP. PEI is specifically favored because of its positive charge, whereas PIP shows great tunability while synthesizing PA-NF membranes.^55,56^ Sodium dodecyl sulfate (SDS) is the most commonly used surfactant, with most of its data distribution centered at 0.01 wt %. SDS facilitates better reaction control between the amine-based monomer and TMC by forming a self-assembled network at the water/organic solvent interface.^57^ This allows for the formation of a compact PA layer with uniform pore sizes. Studies often used acid acceptors such as sodium carbonate and sodium phosphate. These acceptors modulate the diffusive flux of amine monomers, resulting in the formation of thin PA layers with structural homogeneity, allowing for stable membrane performance.^58^ Over 88% of the data set consists of studies about standard IP, whereas 11.5% of the data points pertain to a relatively new method known as reverse interfacial polymerization (RIP). Researchers have found several advantages in RIP over IP, the biggest of which is the existence of residual positively charged amine monomers on the surface after membrane synthesis, allowing for better Li/Mg separation performance.^59^

The data for water permeability and Li/Mg selectivity were collected and utilized to train four ML models (i.e., RF, XGBoost, CatBoost, and LightGBM). The prediction accuracy for the ML models was evaluated using MAE, RMSE, and *R* ^2^. The hyperparameters used to build the final ML models were obtained using Bayesian optimization. The ML model performance for predicting water permeability and salt selectivity is shown in Figure 1. CatBoost showed the best performance while predicting permeability (*R* ^2^ = 0.66), and Li/Mg salt selectivity (*R* ^2^ = 0.65). The MAE errors are ∼1.5 and ∼11%, while the RMSE values are ∼2.5 and ∼17.5% for permeability and Li/Mg selectivity, respectively (Table 1). Only minor performance losses were observed in the model built without using membrane properties. This was done to check the predictive performance of ML models using synthesis conditions only.

**Figure 1:**
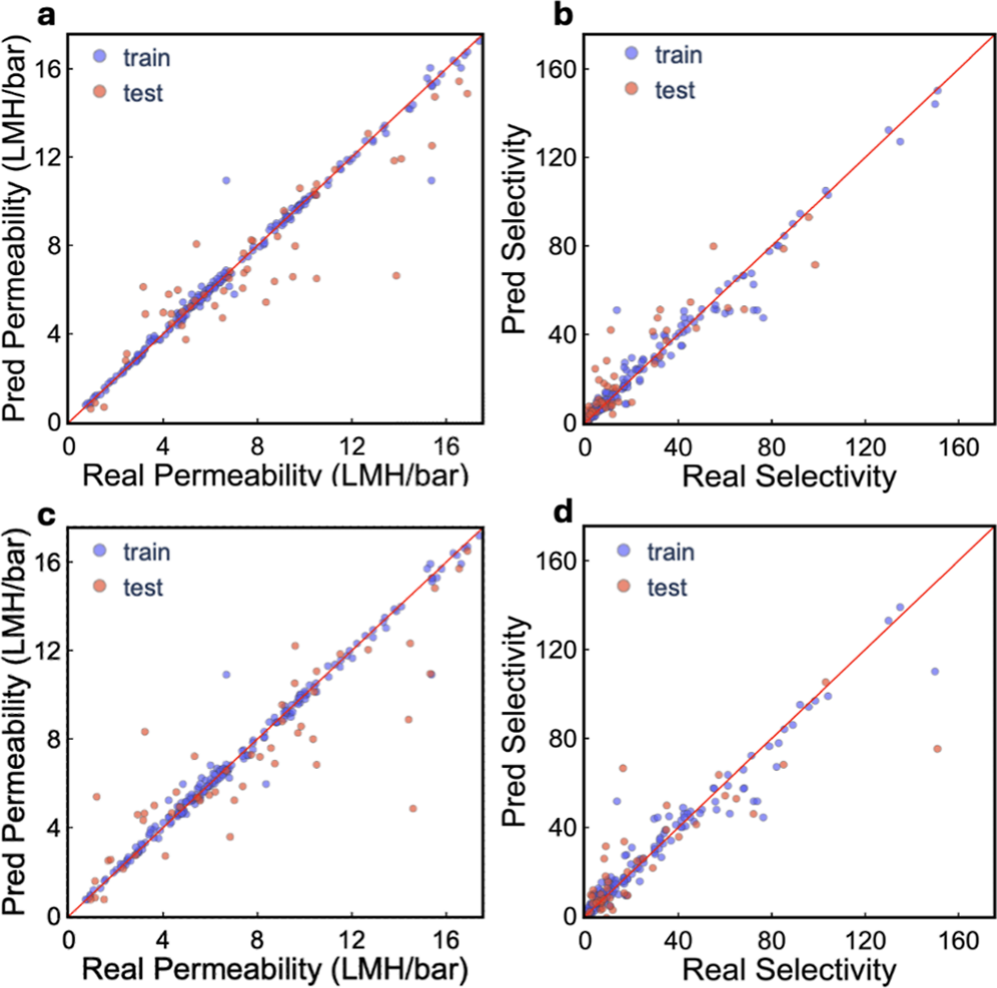
CatBoost-based ML models to predict membrane performance using (a,b) the entire data set, and (c,d) without membrane properties.

**Table 1:** ML Model Performance

		with membrane properties				without membrane properties			
		RF	XGBoost	Catboost	LightGBM	RF	XGBoost	Catboost	LightGBM
permeability (LMH/bar)	MAE	1.72 ± 0.27	1.81 ± 0.11	1.43 ± 0.25	2.40 ± 0.22	1.76 ± 0.27	1.82 ± 0.14	1.45 ± 0.26	2.19 ± 0.30
	RMSE	2.73 ± 0.57	2.28 ± 0.50	2.42 ± 0.25	3.29 ± 0.34	2.73 ± 0.57	2.88 ± 0.53	2.47 ± 0.57	3.17 ± 0.55
	*R* ^2^	0.61 ± 0.10	0.58 ± 0.11	0.66 ± 0.09	0.41 ± 0.06	0.60 ± 0.11	0.58 ± 0.11	0.65 ± 0.09	0.40 ± 0.12
Li/Mg selectivity	MAE	11.66 ± 2.76	12.25 ± 2.23	11.21 ± 2.26	14.82 ± 2.76	11.86 ± 2.69	12.30 ± 3.10	12.08 ± 2.68	14.97 ± 3.306
	RMSE	19.66 ± 2.79	19.19 ± 3.23	17.58 ± 2.26	22.85 ± 3.88	19.16 ± 4.85	19.19 ± 3.23	19.25 ± 3.55	22.29 ± 5.03
	*R* ^2^	0.57 ± 0.12	0.58 ± 0.11	0.65 ± 0.08	0.42 ± 0.10	0.57 ± 0.14	0.58 ± 0.14	0.64 ± 0.10	0.41 ± 0.08

Data leakage occurs when information from the training data set unintentionally influences the model, leading to an overestimation of predictive performance. Jeong et al. have demonstrated the issue of data leakage causing a falsely high prediction accuracy in NF and RO membranes.^46^ In our investigation, researchers within the same study frequently varied MLR as an experimental parameter (i.e., 1, 5, 10, 20, 50) when reporting water permeability and salt selectivity. We showcase the variation in *R* ^2^, MAE, and RMSE for the CatBoost model for water permeability and salt selectivity under these conditions (Table S4, Supporting Information). It can be observed that the model performs much better compared to the reported ML model in this case. To mitigate this, the authors chose only one data point from publications where multiple values of permeability and selectivity were reported for varying MLR, ensuring a more realistic and generalizable model.

### 3.2 Feature Analysis Using XAI

It is important to ensure that the knowledge obtained by the ML model is consistent with the fundamental understanding of design and synthesis of PA-NF membranes for Li/Mg separation. To validate this, we investigated the understanding gained by the ML models using XAI tools such as SHAP and PDP analyses. Figure S4 (Supporting Information) presents the SHAP plots that show the contributions of key features toward the prediction of permeability and selectivity. These plots reveal that membrane properties and the synthesis conditions play a key role in determining membrane performance.

#### 3.2.1 Membrane Properties

To show the impact of membrane properties on the permeability and selectivity, we developed SHAP dependence plots for water contact angle (°), zeta potential (mV), and pore size (Å). Figure 2a,d show the effect of water contact angle on water permeability and selectivity. All the membranes possess a hydrophilic surface (water contact angle is less than 90°), which is considered ideal for water permeability. However, an increase in contact angle within the subrange has a negative impact on water permeability due to enhanced hydrophobic character, which increases the resistance for water transport through the membrane.^50^ On the other hand, the SHAP dependence plot for selectivity shows a sparse distribution throughout the data set range, which indicates that water contact angle has no clear impact on the selectivity of the membrane. Figure 2b,e show the impact of zeta potential on permeability and Li/Mg selectivity, respectively. Selectivity improves significantly as zeta potential increases beyond 0 mV, suggesting that a positively charged surface allows for easier permeation of Li^+^ ions as compared to Mg^2+^ ions. This is due to the dielectric and Donnan effects, which result in stronger repulsion forces faced by divalent ions (Mg^2+^) as compared to monovalent ions (Li^+^).^54,60,61^ Tuning membrane surface charge is thus a useful strategy to improve the selective behavior of membranes to achieve separation of Li and Mg. However, in the case of permeability, negative values of zeta potential are more favorable for water transport due to the formation of a hydration layer under negatively charged conditions, promoting water passage.^62,63^ In Figure 2c,f, the influence of pore size on membrane permeability and selectivity was illustrated. To understand the influence of pore size on Li/Mg selectivity, we need to consider two major phenomena, i.e., dehydration energy and size exclusion. Mg (−1830 kJ/mol) requires much more energy to shed its hydration shell as compared to Li (−475 kJ/mol), which favors Li transport through NF membrane pores. However, ions can partially strip their hydration shells to force a passage through membranes. Mg possesses a hydration radius of 4.28 Å and a Stokes radius of 3.47 Å, whereas Li possesses a hydration radius of 3.84 Å and a Stokes radius of 2.40 Å.^64^ Peng et al. suggested that the mean pore radius of the membrane pore sizes should lie between the hydration radius of Mg and the Stokes radius for Li (i.e., between 2.4 and 4.28 Å) for exceptional separation performance of both the elements.^65^ However, as per Figure 2f, there is a variation of SHAP values from negative to positive for Li/Mg selectivity within this subrange, possibly due to material-specific interactions with Li and Mg. This warrants a deeper investigation into pore size control of NF membranes to synthesize membranes with high selectivity. The broader trend, however, still shows that membrane permeability is compromised when designing selective membranes.

**Figure 2:**
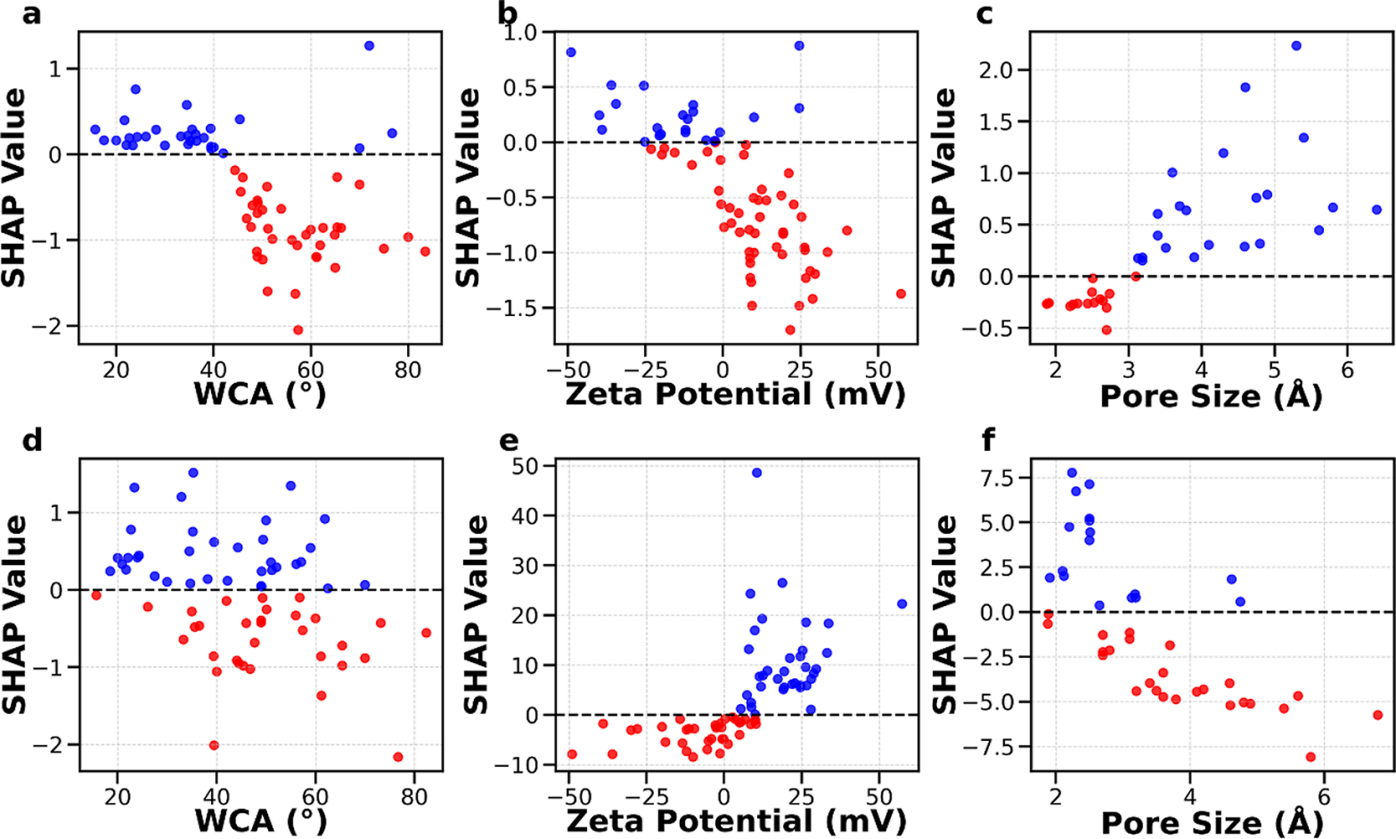
SHAP analysis of membrane properties for water permeability: (a) water contact angle, (b) zeta potential, (c) pore size; SHAP analysis of membrane properties for Li/Mg selectivity: (d) water contact angle, (e) zeta potential, (f) pore size. Blue and red colors represent positive and negative SHAP values, respectively.

#### 3.2.2 Impact of Synthesis Conditions

To gain a deeper understanding of the impact of synthesis conditions on membrane permeability and selectivity, we used our data set trained on CatBoost model to construct single-variable and multivariable PDP (Figure 3). In Figure 3a,b, the impact of monomer A1 and TMC concentration on water permeability and salt selectivity is illustrated. An increase in the concentration of monomer A1 and TMC in the synthesis solution results in a compact polymer network, which inhibits water permeability and promotes Li/Mg salt selectivity. Zhu et al. also found a linear increase in active layer thickness with increasing concentrations of amine-based monomer.^66^ This increases the mass transfer resistance across the membrane, having negative effects on water permeability.^67^ An excess of monomer A1 compared to TMC also results in unreacted monomers on the membrane surface, enhancing its positive charge and promoting Donnan exclusion, which directly contributes to membrane selectivity.^55^ Low concentrations of monomers can lead to a lesser degree of cross-linking and larger pore sizes, which have a negative impact on the selective behavior of the membrane.^68^ Figure 3c,f show the effect of polymerization time on membrane permeability and selectivity. As the polymerization time increases, monomers A1 and TMC get more time to diffuse and react, enhancing the degree of cross-linking and thus generating a denser active layer. The improved degree of cross-linking decreases membrane permeability while improving Li and Mg separation performance.^69^ Figure 3d,e,g,h show the impact of the variation of heat curing time and heat curing temperature. Heat curing is an essential step in the fabrication of NF membranes since it facilitates polymerization between unreacted monomers and removal of excess solvents. Longer heat curing times are associated with the formation of a denser PA layer since it promotes cross-linking, which improves membrane selectivity.^70^ The trends regarding curing temperature are less consistent. Researchers have found that even though heat-curing temperature increases the rate of polymerization, it can also damage the active layer of the NF membrane, resulting in surface defects.^71^

**Figure 3:**
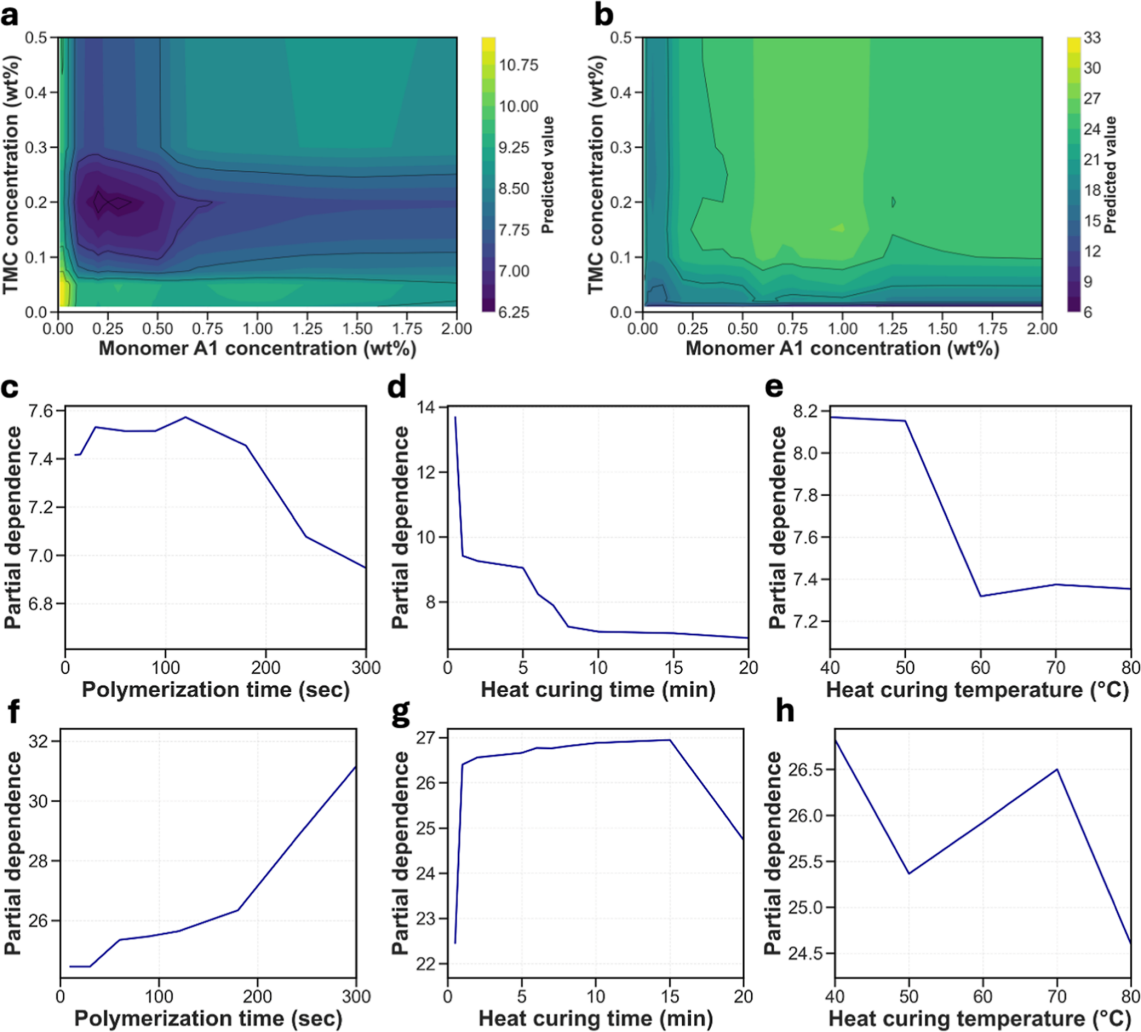
PDP analysis for membrane performance. PDP showing the effect of monomer A1 concentration (wt %) as a function of TMC concentration (wt %) on (a) water permeability and (b) Li/Mg selectivity. In (a,b), lighter regions correspond to feature values associated with higher membrane performance. PDP of (c) polymerization time, (d) heat curing time, and (e) heat curing temperature for water permeability. PDP of (f) polymerization time, (g) heat curing time, and (h) heat curing temperature for Li/Mg selectivity.

The results obtained in Figures 2 and 3 highlight the general trends and the stark contrast in how these synthesis conditions and membrane properties impact water permeability and selectivity. Factors that are conducive to membrane permeability negatively affect selectivity and vice versa. These results also highlight the significant influence of monomer A1 on membrane performance, underscoring the need to develop innovative material screening strategies to enhance design efficiency.

### 3.3 Potential Monomer Screening and Experimental Validation

Based on the SHAP values of input MFs, we identified 15 functional groups having a positive influence on membrane permeability and 12 functional groups having a positive influence on membrane selectivity. As shown in Figure S5a (Supporting Information), the presence of hydrophilic carbonyl and sulfonate groups can contribute positively to water permeability.^19^ As per Figure S5b (Supporting Information), the presence of positively charged nitrogen centers can improve the surface charge of the membrane. This can positively impact Li and Mg separation in membranes, directly contributing to its selectivity.^72^ Additionally, the presence of primary, secondary, and tertiary amines is necessary since it forms the polymeric backbone during the synthesis of PA-NF membranes. Using these features, we constructed a reference MF that was used to screen monomers from the monomer database using the Tanimoto coefficient.^73^ We selected the top 20% of the monomers from this list for further investigation.

For the second stage of screening, we used radius of gyration, topological polar surface area (TPSA), BalabanJ, sphericity index, eccentricity, inertial shape factor, maximum partial charge, and logarithm of partition coefficient (logP) as MDs of choice.^35^ Importance and correlation analyses on the SHAP values of the MDs were carried out for different purposes. Importance was calculated to quantify the contribution of the MD to the ML models’ predictive performance (Figure 4a,c).^29^ On the other hand, correlation was calculated to evaluate the directionality and strength of the relationship between the SHAP values of the MD and membrane performance (Figure 4b,d). The SHAP values of the MD that are positively correlated to membrane performance are more desirable and vice versa for negative features (Figure S6, Supporting Information).

**Figure 4:**
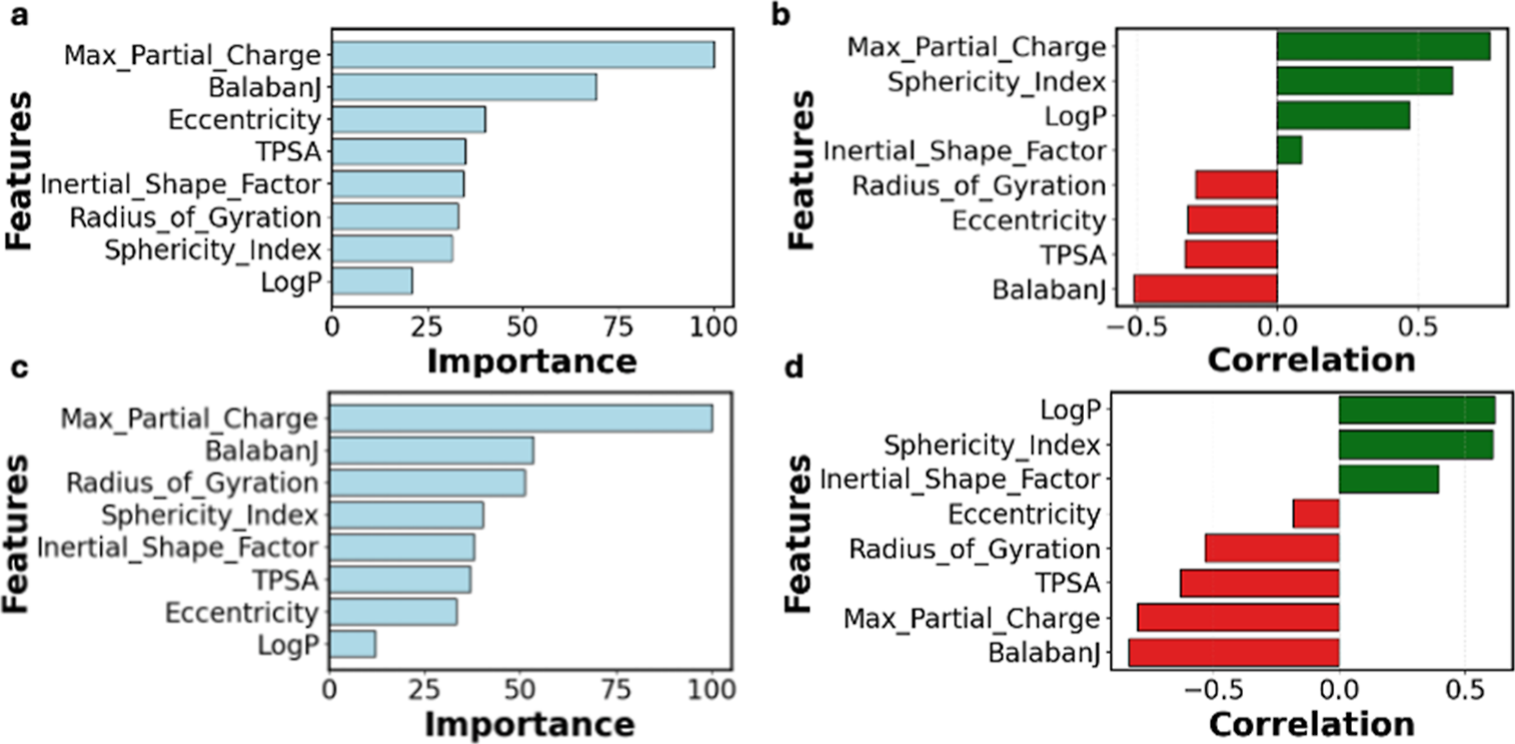
SHAP Analysis of MDs for membrane performance. (a,b) correspond to water permeability, and (c,d) to Li/Mg selectivity. Green bars in correlation plots imply a positive correlation, whereas red bars imply a negative correlation.

The separation performance of Li and Mg can be traced to the effect of these descriptors on membrane permeability and selectivity. Max_Partial_Charge is related to the reactivity of the monomer, wherein a greater value is associated with the electron richness of the molecule. IP is a reaction where the amine monomer reacts with the acyl chloride monomer at the water–hexane interface. Increasing Max_Partial_Charge can accelerate the reaction, resulting in the formation of a loose, porous structure that negatively impacts solute separation performance. It is desired to have a slower controlled reaction such that a uniform membrane layer is formed.^57^ This can enhance size sieving effects, which allows for better separation performance. TPSA is associated with increasing molecule complexity due to the presence of reactive sites, resulting in the formation of membranes with loose structures. Thus, TPSA also has a negative contribution toward the separation performance of the membrane.^35^

After the correlation and importance of the features were calculated, we evaluated the feature impact by multiplying the median correlation and importance obtained across 8 seeds. This would ensure that both directionality and contribution of the MDs are considered when screening potential monomers. We scale the MD of the potential monomers by the feature impact for both permeability and selectivity and add those values to receive a screening score (Figure 5). Monomeric candidates having a higher score would have more desirable MDs, taking both permeability and selectivity into account. Thus, we can streamline the screening process and identify materials that are more likely to succeed as potential monomers to synthesize high-performance NF membranes for Li separation. We can potentially tweak the outcomes from this process and find monomers that either showcase more selectivity or more permeability by assigning different weights to both the ML models while calculating the screening score. This will ensure more desirable outputs are favored. The top 20% of the monomers having the highest screening factor were checked for their synthesis feasibility.

**Figure 5:**
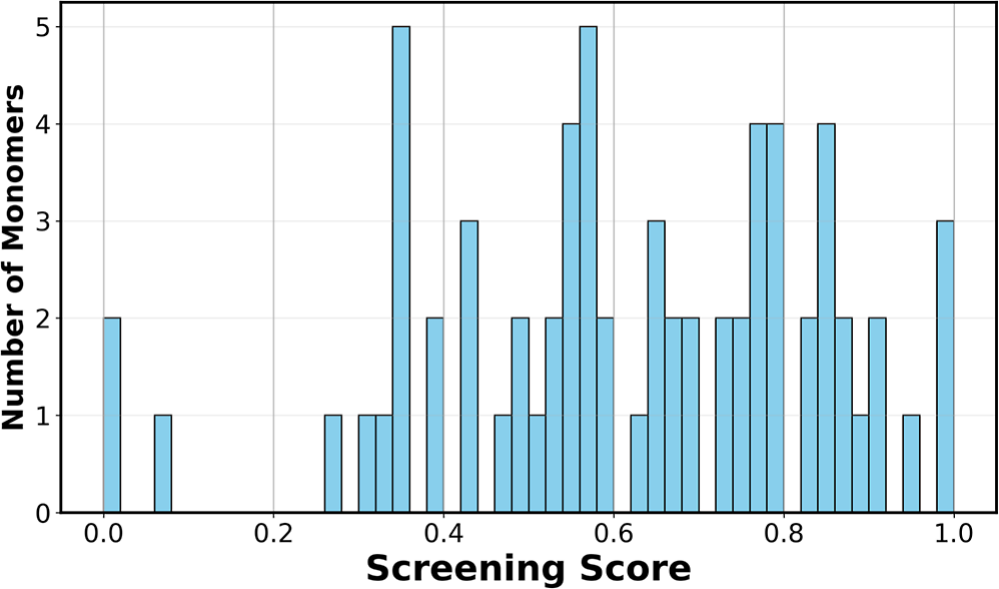
Screening score of the potential monomer candidates.

The final stage of the screening process involves the calculation of SAS.^74^ The SAS contains fragment contributions and complexity penalty as two major components. The fragment contributions present in molecules that are more difficult to synthesize are scored higher as compared to fragments of commonly known molecules that are easier to synthesize. Complexity penalty assigns a penalty to large rings, ring fusions, and overall molecular size. After the SAS was calculated for our screened monomers having the highest screening factor, we report five monomers with low SAS to find the monomer candidates that show potential for membrane synthesis (Figure 6).

**Figure 6:**
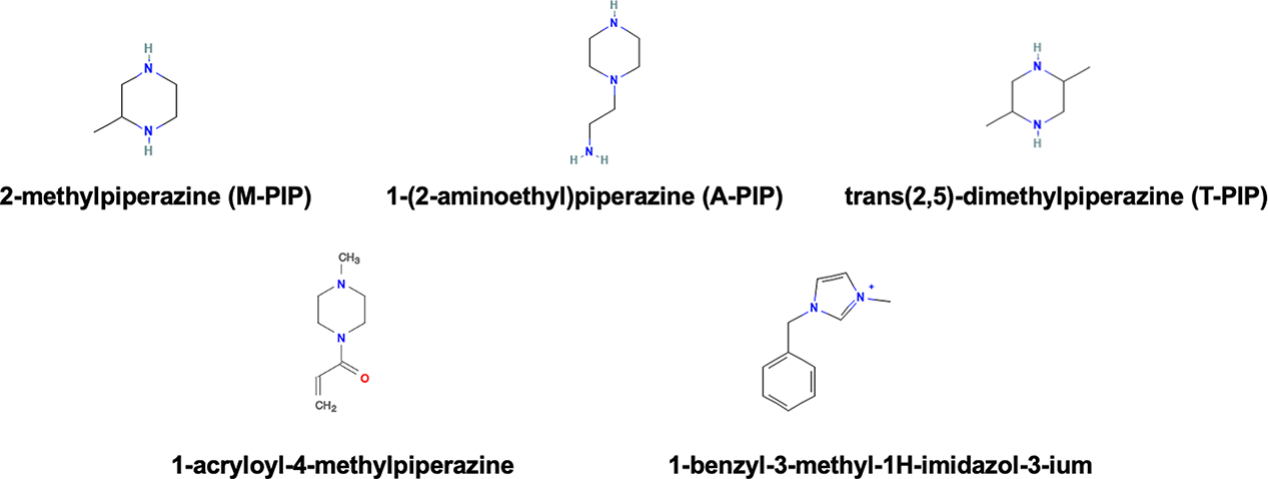
Monomer candidates screened using the ML model.

As observed, all these monomers have nitrogen centers, which are essential for the IP reaction with TMC. In the case of 1-acrylol-4-methylpiperazine, it has a carbonyl group on its surface, which can enhance water permeability. A positively charged nitrogen is present in 1-benzyl-3-methyl-1*H*-imidazole-3-ium that can impart a positive charge to the membrane surface, resulting in the improvement of charge screening effects, promoting better separation of Li and Mg. Out of the five reported monomers, membranes were synthesized using three candidates: A-PIP, M-PIP, and T-PIP. Among these, A-PIP and M-PIP are superior to PIP in terms of membrane permeability and salt selectivity at similar synthesis conditions (Figure 7). Specifically, M-PIP exhibited a greater membrane flux as compared to PEI, although it has lower salt selectivity, whereas A-PIP surpasses PEI on both water permeability and salt selectivity. T-PIP, however, did not demonstrate stable performance during membrane testing. These results highlight the promise of the screening approach but also underscore the importance of experimental validation to confirm the practical viability of the selected monomer candidates.

**Figure 7:**
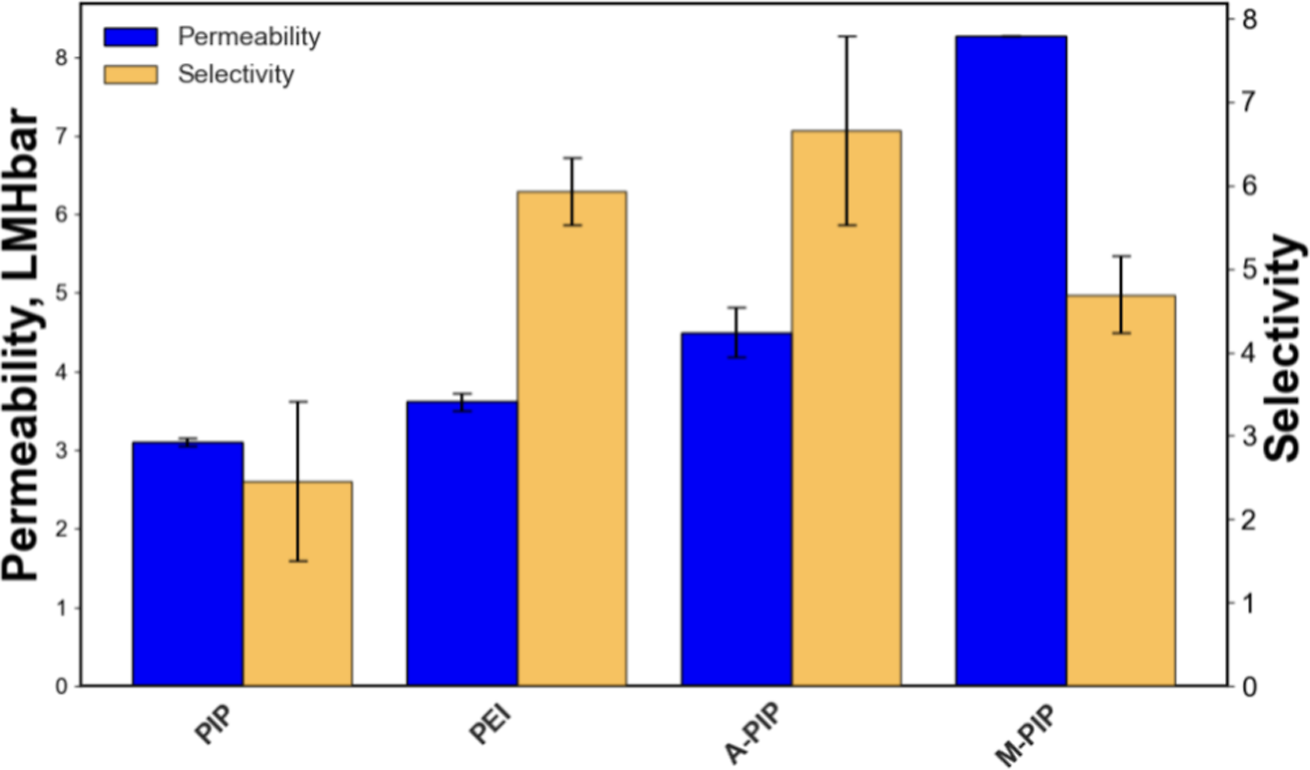
Performance characteristics of screened monomers.

## 4 Implications and Future Perspectives

In this study, we present the design considerations and a novel material screening approach to synthesize high-performance NF membranes for Li separation. We conduct a comprehensive ML analysis to relate the input synthesis conditions, membrane properties, and operating conditions to membrane performance and elucidate the underlying principles governing the separation of Li and Mg using PA-NF membranes. XAI (i.e., PDP and SHAP analysis of the synthesis conditions) revealed the importance of amine and chloride monomer concentrations, reaction time, heat curing time, and heat curing temperature on membrane performance. Additionally, membrane properties such as pore size and zeta potential are critical to understanding the water transport and separation behavior of Li from Mg in NF membranes. These findings highlight the potential for research on positively charged materials that increase the surface charge and techniques to achieve a sharp pore size distribution, potentially improving the outcomes for Li and Mg separation. In general, there was an inverse relation between parameters favoring permeability compared to those favoring selectivity. This is in line with the literature on the permeability-selectivity trade-off commonly observed in polymeric membranes. Researchers need to make use of advanced toolkits, such as those lying within the inverse-design umbrella, to tackle this trade-off. In that context, we developed a high-throughput virtual screening pipeline that leverages the physical and chemical descriptors used to represent monomers. For this purpose, we used a 3-step setup that combines MFs, MDs, and SAS to screen candidates for Li–Mg separation. The goal was to find the promising candidates from a given list by maximizing their physical and chemical characteristics with the help of the Tanimoto coefficient and our in-house derived screening score. We reported five candidates in our study using this methodology and tested the performance of three candidates against PIP and PEI, which are commonly used materials in the literature. Two candidates had better permeability and selectivity as compared to PIP at similar experimental conditions, showing the potential of this screening process.

In an ideal scenario, models should be able to learn from high-quality, clean, labeled data encompassing a diverse chemical space and experimental conditions that are representative of real-life scenarios. However, models trained for material screening in membrane technology are often trained on smaller data sets, where such a type of data diversity is not available. This raises concerns regarding the transferability of these models across different chemical spaces and experimental conditions, which are unseen in the data set. It becomes essential to test and validate the performance of novel materials screened with the help of ML models in environmentally relevant conditions to alleviate these concerns. Further, the quality of data in experimentally derived data sets is prone to noisy observations due to variability in measurement techniques across laboratories, measurement device inaccuracy, and random human error. Using tools such as WebPlotDigitizer to mine data from graphs can reduce the inconsistencies in data collection as compared to human observations, however its results are dependent on graph quality, image resolution, and human accuracy. These tools also often lack the provision to measure uncertainty using error bars. Data needs to be rigorously checked, preprocessed, and post-training cross-validation techniques need to be implemented to reliably assess model performance and ensure its robustness while minimizing bias. Even though our model shows satisfactory performance over a wide range of parameters, there are several ways in which it can be improved. The most important step is to find methods to represent categorical features, such as additives in the aqueous phase and the additive layer, in a similar fashion to the monomers used in the study. This would ensure that we can capture chemical and physical details from those modifications, which can be further used to enhance the screening process. Furthermore, there were only 12 types of different amine monomers present in the data set. This results in variations in how the ML model captures the chemical fingerprints and MDs while carrying out the screening process (Figures S7 and S8, Supporting Information). Expanding the data set with more monomers and data points will help in improving the chemical diversity of the data set.

Material screening is only the first step in the design hierarchy required to synthesize high-performance membranes. Inverse design has revolutionized materials design and discovery by fundamentally flipping the research paradigm by selecting desired properties of the material and working backward to identify ideal candidates.^75,76^ Generative ML allows for autonomous design of new chemicals based on previously trained data. Researchers can produce hypothetical candidates with user-defined criteria and incorporate constraints into the generative process targeted to optimize materials performance, thereby accelerating materials discovery. Ultimately, the final performance of membranes is dependent on the close interplay of synthesis conditions and the resultant membrane properties. A deep understanding of these relationships is essential to carefully balance the “permeability-selectivity” trade-off. Being reliant on the traditional trial-and-error approach to do this is costly and time-consuming, thus requiring alternate approaches to navigate this infinite search space consisting of thousands of monomer/synthesis condition combinations. Active learning and Bayesian optimization are ML strategies that employ surrogate models to predict the properties of unexplored materials and guide experiments in a way that maximizes learning, reduces model uncertainty, and optimizes outcomes.^19,77^ This approach is particularly useful since we can gain information with limited experimental resources. Researchers have relied on using Pareto optimization to identify a set of materials (“Pareto Fronts”) to find balanced performance among the chosen metrics.^78^ Even though Pareto optimization provides an interesting solution to the multiobjective design problem, its success hinges on the predictive accuracy of the underlying ML models.^79^ Research advances are most likely to occur when experimental work is integrated with ML and computational approaches. This will transform the traditional discovery process and allow efficient exploration of large chemical spaces, leading to the development of a superior class of NF membranes.

## Supplementary Material





## Data Availability

All the data used in the study are presented in the Supporting Dataset.

## Notes

Variables used for data set construction; morgan fingerprint and molecular descriptors used for this study; hyperparameter ranges of the ML model; performance metrics for the CatBoost model when data leakage occurs; permeability–selectivity plot for the data set, data characteristics for numerical features; data description for categorical features; SHAP plots for permeability and Li/Mg selectivity; chemical bonds with positive contribution to membrane permeability and Li/Mg selectivity; SHAP plots relating molecular descriptors to membrane selectivity; SHAP plots showing the variation in correlation and importance for multiple seeds (PDF)
Supporting Dataset (XLSX)
